# General characteristics of relative dispersion in the ocean

**DOI:** 10.1038/srep46291

**Published:** 2017-04-11

**Authors:** Raffaele Corrado, Guglielmo Lacorata, Luigi Palatella, Rosalia Santoleri, Enrico Zambianchi

**Affiliations:** 1 CNR, Institute of Atmospheric and Climate Sciences, Lecce, I-73100, Italy; 2 CNR, Institute of Atmospheric and Climate Sciences, Rome, I-00133, Italy; 3 University of Naples ”Parthenope” and CONISMA, Naples, I-80143, Italy

## Abstract

The multi-scale and nonlinear nature of the ocean dynamics dramatically affects the spreading of matter, like pollutants, marine litter, etc., of physical and chemical seawater properties, and the biological connectivity inside and among different basins. Based on the Finite-Scale Lyapunov Exponent analysis of the largest available near-surface Lagrangian data set from the Global Drifter Program, our results show that, despite the large variety of flow features, relative dispersion can ultimately be described by a few parameters common to all ocean sub-basins, at least in terms of order of magnitude. This provides valuable information to undertake Lagrangian dispersion studies by means of models and/or of observational data. Moreover, our results show that the relative dispersion rates measured at submesoscale are significantly higher than for large-scale dynamics. Auxiliary analysis of high resolution GPS-tracked drifter hourly data as well as of the drogued/undrogued status of the buoys is provided in support of our conclusions. A possible application of our study, concerning reverse drifter motion and error growth analysis, is proposed relatively to the case of the missing Malaysia Airlines MH370 aircraft.

Ocean dynamics are characterized by a very broad range of scales of motion. This corresponds to a large number of multiscale dynamical features which are generally present in the different ocean sub-basins: from large scale gyres and boundary currents to mesoscale eddies, down to the submesoscale processes. They are responsible for physical transport in the ocean, whose understanding is crucial for a number of important applications^1^,^2^. Advection and diffusion in the ocean have been intensively studied over the past decades by using Lagrangian instruments^3^,^4^. Observations on trajectory pair dispersion^5^, which is the focus of the present work, have a more recent history^6^. Ad hoc experiments based on the release of drifter pairs or clusters have been organized in the Nordic Seas, in the Atlantic Ocean and in the Mediterranean Sea^2^,^7^. An extremely large deployment of drifter clusters was performed in the Gulf of Mexico to investigate the spreading of oil in the aftermath of the Deepwater Horizon spill in 2010^8^,^9^.

The main goal of this work is to present a study of the relative dispersion properties in the ocean upper layer, through the analysis of surface drifter data from NOAA Global Drifter Program^10^, described in detail in the Data section. We focus our attention on relative, or two-particle, dispersion since this can give valuable information about flow structures over many scales of motion, unlike absolute, or one-particle, dispersion which mainly depends on the large-scale features of the system. In Lagrangian modelling studies, for example, is generally simpler to simulate correctly one trajectory diffusion on large scales rather than two trajectory dispersion at non asymptotic scales. Relative dispersion properties are usually expressed in terms of scaling laws. These scaling properties have been analyzed, so far, for isolated ocean basins, and not always using optimal techniques. We propose a Lagrangian data analysis based on the computation of the Finite-Scale Lyapunov Exponent (FSLE), described in detail in the Methods section, which provides, on one hand, an objective measure of scaling exponents and physical parameters, and, on the other hand, offers the opportunity to form a global overview of relative dispersion scale-dependent characteristics of the ocean surface layer.

The large-scale circulation of the upper ocean is mainly driven by wind stress. Therefore, we decided to partition the global ocean surface dynamics on the basis of the climatological maps of this variable into 11 macro-areas^11^ characterized by different large-scale and near-surface dynamics, see Fig. 1. Of course, other criteria can be considered for the ocean partition, but we think our choice is a good compromise between dynamical and geographical arguments. The results we present provide a measure of average dispersion properties at basin scale (for each partition element), which, in a Lagrangian modelling perspective, represent the benchmark against which numerical simulations should be tested. A list of acronyms used in this work is reported in Table 1.

## Results

**Figure 1:**
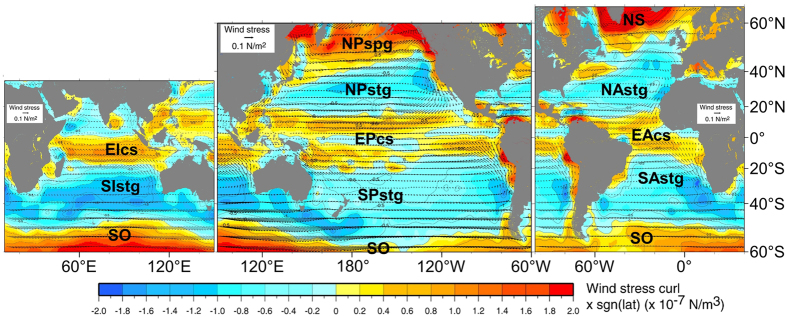
Surface ocean dynamics partition utilized in this work, based on the annual mean wind stress (vectors) and wind-stress curl (color shading, multiplied by −1 in the Southern Hemisphere). Based on, and modified from Figs S9.3a, S10.2a and S11.03a of the book “Descriptive physical oceanography: an introduction” by Talley *et al*., Academic Press (2011)^11^, reproduced with permission. For a list of acronyms used in the figure and in the body of the paper see Table 1.

**Figure 2:**
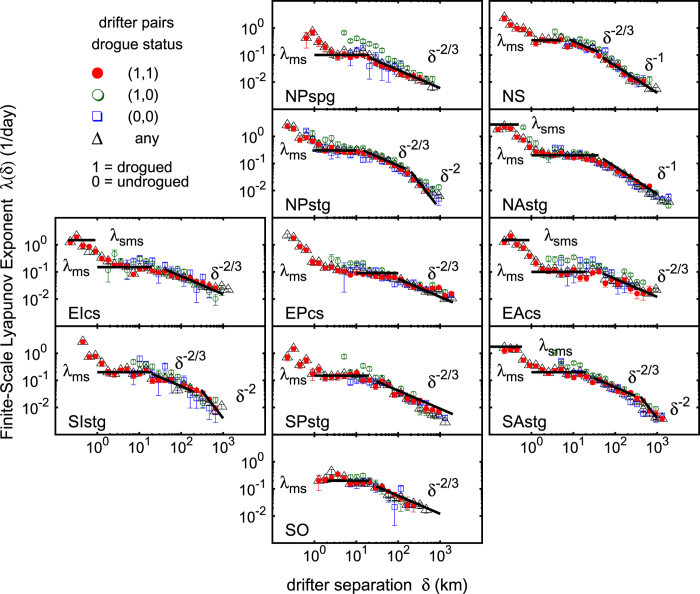
FSLE computed on the Global Drifter Program data set subdivided in eleven ocean basins (see Fig. 1). Colors are associated to the drogue status of the SVP drifter pairs: red = drogued (1,1); green = drogued/undrogued (1,0); blue = undrogued (0,0); black = any status. The FSLE scaling exponent, *λ(δ*) ~ *δ*^−*β*^, corresponds to: Richardson’s dispersion (*β* = 2/3), shear/ballistic separation (*β* = 1), and standard eddy-diffusion (*β* = 2). Exponential separation rates, i.e. *λ(δ*) ≃ constant, are indicated as *λ*_*ms*_, for the mesoscales, and as *λ*_*sms*_, for the submesoscales (when resolved). Maximum initial separation allowed: Δ = 10 km. Amplification ratio 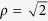. The FSLE *λ(δ*) is inversely proportional to the characteristic time scale of dispersion at scale *δ*. Panel generated with GNUPLOT 5.0 (Williams, T. and Kelley, C., 2011; Gnuplot 5.0: an interactive plotting program; URL: http://gnuplot.info).

The results of the FSLE analysis of the drifter data sets, divided by geographic area according to Fig. 1, are shown in Fig. 2. Since the presence of the drogue in the SVP type of buoy is supposed to guarantee the correct Lagrangian tracking of the ocean surface currents, and since a buoy may lose the drogue during its motion, we investigated the sensitivity of the FSLE computation to all possible combinations occurring for a drifter pair: both drifters with drogue attached, one drifter drogued and the other one undrogued, both drifters without drogue attached, or any of the previous cases (i.e. no check of the drogue status). This is clearly shown by the different symbols in Fig. 2, and will be briefly discussed below.

**Figure 3:**
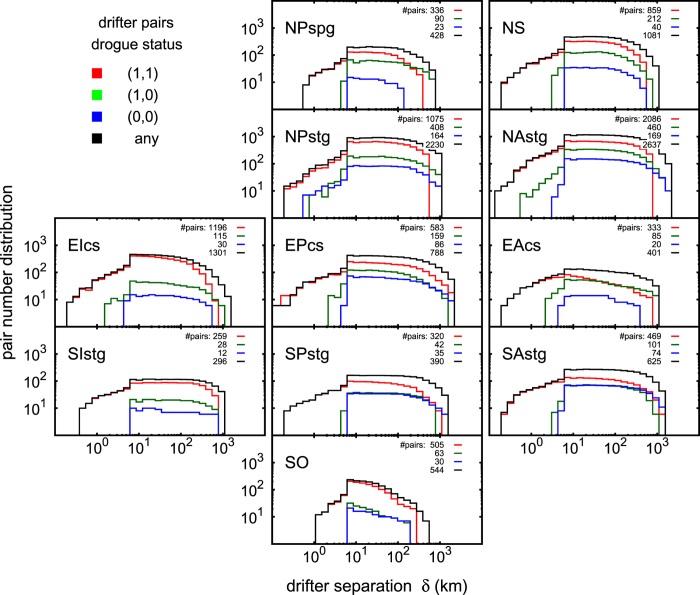
Drifter pair statistics of the FSLE analysis. Colors are associated to the drogue status of the drifter pairs. The numbers plotted on the top right corner of each panel are the total number of pairs analyzed for each drogue status: drogued (1,1); drogued/undrogued (1,0); undrogued (0,0); any status. The statistics is also determined by the filter imposed to the maximum allowed initial separation of the drifters (Δ = 10 km). Panel generated with GNUPLOT 5.0 (Williams, T. and Kelley, C., 2011; Gnuplot 5.0: an interactive plotting program; URL: http://gnuplot.info).

Statistics of the analyzed drifter pairs, case by case, are presented in Fig. 3. The sample size is essentially determined by the filter imposed to the maximum initial separation allowed, i.e. only drifter pairs with initial separation ≤Δ = 10 km are taken into account, which is a good compromise between maximizing the number of pairs and keeping the FSLE analysis within reasonable physical constraints. The amplification factor between neighboring scales is always set to 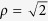. In all plots, points with a pair number less than 6 are ruled out.

**Figure 4:**
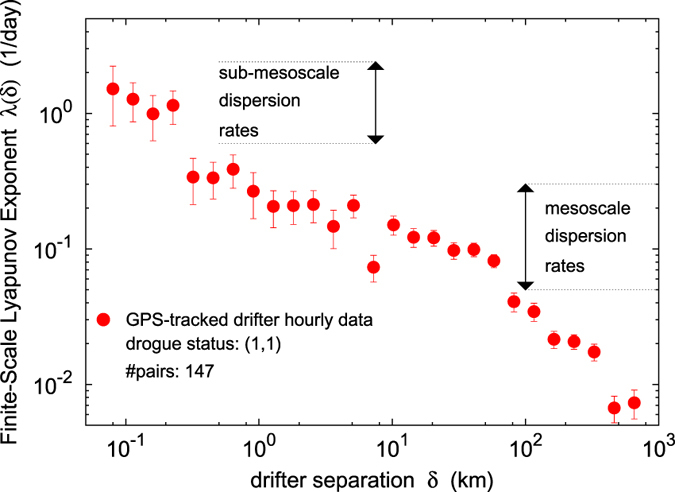
FSLE computed for the GPS-tracked drifter data set. The relative dispersion rates measured by the FSLE for the sub-mesoscale (below ~1 km) range are significantly higher (about an order of magnitude) than for the mesoscale range (~10–100 km). GPS-tracked drifter positions are affected by ~10^−5^ degree errors. Only drifter pairs with drogue status (1,1) were considered. Maximum initial separation allowed: Δ = 10 km. Amplification ratio 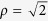. The data refer to the global ocean (with no geographic constraint). Color map generated with GNUPLOT 5.0 (Williams, T. and Kelley, C., 2011; Gnuplot 5.0: an interactive plotting program; URL: http://gnuplot.info).

It is worth noticing that Fig. 2 displays also results relative to the submesoscale range, i.e. scales below ~1 km. On the other hand, ARGOS-tracked drifter data may be affected by significantly large errors, comparable to such scales. This suggests to take relative dispersion results at scales lesser than 1 km with extreme caution, just as a qualitative indication under the assumption (not verified, R. Lumpkin, personal communication) that location errors may not be totally uncorrelated for close simultaneous particle pairs. Therefore, in order to robustly assess the presence of local submesoscale separation mechanisms, we replied the FSLE analysis on high resolution GPS-tracked drifter hourly data, so as to better investigate the relation between mesoscale and sub-mesoscale relative dispersion rates. The results of this analysis are shown in Fig. 4; given the low number of suitable GPS-equipped drifter pairs (149), the analysis was carried out for the whole ocean.

**Figure 5:**
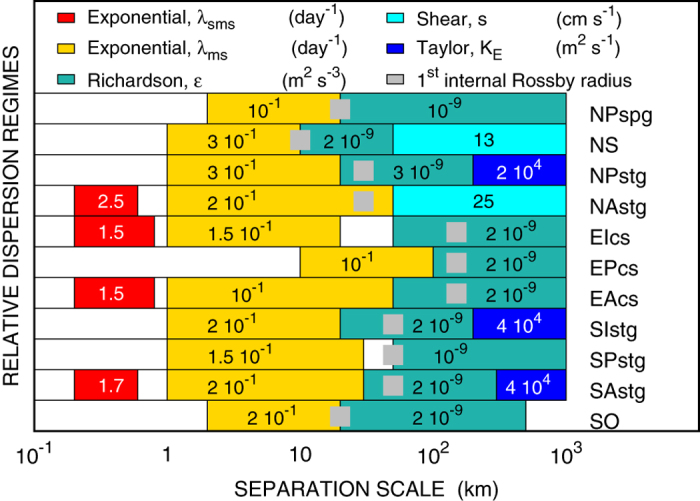
Major dispersion regimes observed in the 11 sub-basins of the ocean partition. Colors refer to the type of relative dispersion: red = submesoscale exponential, yellow = mesoscale exponential, water green = Richardson, cyan = Shear/Ballistic, blue = Taylor. Gray squares represent the order of magnitude of the average latitude-depending Rossby radius scale^12^. The numerical values of the physical parameters (exponential separation rates, turbulent dissipation, shear velocity, eddy diffusion coefficients) were obtained by fitting the corresponding scaling laws to the FSLE data, see Fig. 2. Panel generated with GNUPLOT 5.0 (Williams, T. and Kelley, C., 2011; Gnuplot 5.0: an interactive plotting program; URL: http://gnuplot.info).

An important quantity characterizing flow instability, and useful to consider when discussing scaling laws associated to relative dispersion, see Fig. 5, is the first internal Rossby radius of deformation (or, for simplicity, Rossby radius), defined as *R*_*I*_ ~ *N*_*b*_*H*/[*πf(θ*)] (except at the equator) where *N*_*b*_ is the buoyancy frequency, *H* the water column depth and *f(θ*) the latitude depending Coriolis parameter. We refer the reader to Chelton *et al*.^12^ for a detailed study on the geographical variability of the Rossby radius.

We begin our analysis with the northern subtropical gyres (the following considerations refer mainly to pairs characterized by the mutual presence of the drogue, see below). The **NAstg** FSLE spectrum can be divided into three main regimes:

- a short plateau at *δ* < 1 km, hinting at the presence of submesoscale features that significantly enhance the dispersion rate (about one order of magnitude with respect to the mesoscale);
- another plateau between 1 and 50 km, corresponding to mesoscale-driven exponential separation, which begins at a scale close to the Rossby radius (i.e., approximately ≃30 km) and ends at nearly 1 km;
- ballistic/shear dispersion at larger scales, up to *O*(10^3^) km, likely due to the dominant action of the Gulf Stream and its associated recirculations.

Different regimes are found for **NPstg**: the FSLE shows a negative slope for separation below 1 km, even though in this case a submesoscale plateau is not resolved; exponential separation is present between 1 and 10 km; a Richardson-like dispersion between 10 and 100 km; a diffusive regime beyond 100 km.

Dispersion regimes in the equatorial regions have clear similarities in the three oceans: submesoscale dynamics is characterized by a dispersion rate of an order of magnitude larger than the mesoscale, with a clear plateau in EPcs and EIcs, for scale separation of order ≤1 km. At larger scales, **EAcs**, **EPcs** and **EIcs** show two major regimes: a mesoscale plateau and a Richardson dispersion over a large range centered on the Rossby radius.

The FSLE spectra in the southern subtropical gyres, **SAstg**, **SPstg** and **SIstg**, display a rich picture of different regimes: they all carry a mesoscale plateau and a Richardson type of dispersion at larger scales; SAstg and SIstg display also standard diffusion^13^ above 300 km. Our estimate of the eddy diffusion coefficient, of the order O(10^4^) m^2^/s, corresponds to values measured in the ocean with other techniques^14^,^15^,^16^.

With regards to high latitudes, in the **NS** we observed three regimes: exponential separation from 1 to 10 km and a short Richardson scaling between 10 and 50 km, substantially in agreement with the results by Koszalka *et al*.^17^; and a long range of shear/ballistic dispersion up to separations ~1000 km, probably because of the presence of currents responsible for long range spatial correlations, as for the last offshoots of the North Atlantic Drift. A different situation is displayed in the **NPspg**, where a more typical exponential separation/Richardson’s regime scenario, common to other sub-basins, can be assessed.

Finally, the **SO**, characterized by the presence of the Antarctic Circumpolar Current (ACC), displays two main regimes: mesoscale exponential separation; and a Richardson type of scaling up to approximately 800 km. Because of the peculiar anisotropy characterizing the ACC, the meridional (i.e. cross-stream) FSLE was also computed and included in Supplementary Material, Fig. S1. Relative dispersion across the ACC is confirmed to display a standard eddy-diffusion regime, unlike the global FSLE results for the SO which are dominated by the zonal (along-stream) dynamics.

As mentioned above, this discussion was referred to pairs composed of both drogue-equipped instruments. In fact, such a behaviour is also shared by pairs composed of both non-drogue-equipped drifters. Figure 2 shows that only pairs composed of one drifter with and the other without the drogue display a different dispersion. Namely, they are characterized by higher separation rates at scales of the order of 10 to 100 km, and occasionally higher (as in the case of the **EAcs**). Since undrogued drifters are more subject to direct surface wind forcing, we can expect that such pairs can experience an enhanced separation at the atmospheric mesoscales, where atmospheric and oceanic behaviour strongly differ from each other.

## Discussion

The upper ocean circulation is dominated by a high number of subsystems, each of them characterized by its own dynamical features, e.g., boundary currents, gyres, and eddies. Although making inter-comparisons between different regions may seem difficult^18^, we have found that the basic Lagrangian dispersion characteristics are actually shared by most ocean basins. Namely:

- mesoscale exponential separation is present ubiquitously in the whole global ocean, and it is characterized by a *mesoscale Lyapunov exponent* (in broad sense) *λ*_*ms*_ ~ *O*(10^−1^) day^−1^, on scales approximately below the Rossby radius^19^;
- a Richardson regime^5^, on scales comparable to the Rossby radius or larger, appears in all sub-basins and is characterized by an *equivalent* mean turbulent dissipation rate *ε* ~ *O*(10^−9^) m^2^/s^3 ^20,21 everywhere (with the exception of the NAstg);
- the Atlantic northern hemisphere (NAstg and NS) displays the presence of long-range shear/ballistic dispersion, possibly due to the action of the Gulf Stream and its north-eastern extension. In all cases we measured a mean separation velocity *s* of the order of ~*O*(10^−1^) m s^−1^. This is a relatively new result since previous studies on ocean drifter relative dispersion for NAstg^22^ and for NS^17^ did not assess this type of long-range regime. A punctual comparison is not possible essentially because of the different analysis techniques employed to measure drifter pair dispersion, and different data sets considered.
- a standard diffusive regime^13^ in the three major oceans (NPstg, SAstg, SIstg) was observed thanks to the vastness of these basins, with an eddy diffusion coefficient of the order of *K*_*E*_ ~ 10^4^ m^2^ s^−1 ^21. A similar result was found in the SO also, although limited to the cross-stream (meridional) component of the Antarctic Circumpolar Current (see Fig. S1, Supplementary Material). Also in this case, our estimate of the eddy diffusion coefficient (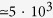 m^2^ s^−1^) is in agreement with previous results found for the Southern Ocean^15^,^23^. As pointed out by Ferrari and Nikurashin (2010)^24^, in conditions of strong zonal advection, meridional diffusivity in the core of the jet may have values much lower than outside the current. A detailed investigation on cross-jet mixing suppression through a direct Lagrangian approach is outside the scope of the present work, since it would require dedicated experiments concentrated in a much smaller geographical domain than we have considered in our global analysis.

With regards to extremely small scales, our results indicate the ubiquitous presence of dispersive mechanisms for trajectory separation of order ~*O*(10^2^) m (i.e. the maximum resolution allowed by the examined data). A definite FSLE plateau, corresponding to exponential separation, is not always detectable, although it is observed in 4 out of 11 cases, characterized by a *sub-mesoscale Lyapunov exponent λ*_*sms*_ ~ *O*(1) day^−1^ everywhere. From the analysis of the GPS-tracked drifter data we can conclude that, regardless the accuracy of the small-scale FSLE plateau, the characteristic rates of dispersion in the sub-mesoscale range are significantly higher than for the mesoscale (about one order of magnitude). Our data are thus compatible with the hypothesis that submesoscale relative dispersion may be locally driven, i.e. dominated by Eulerian structures of the same size as the trajectory separation, with characteristic times not related to larger scale dynamics^7^.

In summary, our results suggest the following:

- In many cases relative dispersion displayed a behavior compatible with a double cascade scenario, i.e. direct enstrophy cascade/inverse energy cascade, below and above the Rossby radius, respectively^25^,^26^,^27^, triggered by baroclinic instability^6^. It is not possible, on the basis of Lagrangian analysis only, to rigorously demonstrate that the observed regimes are the manifestation of quasi two-dimensional turbulence. At most, we can consider them as compatible with such a hypothesis. Moreover, exponential separation may be associated both to non-local chaotic advection (by structures of size larger than the tracer separation with no downscale cascade) and to a *k*^−3^ enstrophy cascade (with characteristic times not depending on the scale). In fact, the inability to clarify these questions is not relevant for most applications and for the scope of this work.
- The issue regarding the shape of the Eulerian energy spectrum at the submesoscales remains open^8^,^9^. New Lagrangian experiments need to be specially planned to investigate these aspects of small scale dispersion with more accuracy, namely to discover if there is an energy gap between meso- and submesoscale ranges or if the two are possibly connected by a continuum of scales.
- An accurate reconstruction of the scaling laws is fundamental to set up kinematic Lagrangian models of turbulent dispersion, conceived in terms of multiscale, deterministic chaotic systems^28^,^29^, for possible future applications to any ocean basin. Other Authors^30^ have also proposed a more standard approach to sub-grid-scale diffusion modelling through stochastic processes.
- The FSLE analysis provides estimates of quantities such as exponential separation rate, turbulent dispersion rate, diffusion coefficient, etc., that are needed to reconstruct the error growth (i.e. trajectory separation), at all available scales of motion, in various Lagrangian applicative studies. We present an example of application to a recent flight accident in the Southern Indian Ocean, discussed in the following section, in which the presumed impact point with its uncertainty area are estimated on the basis of existing ocean drifter backward trajectories and error growth analysis.

### An application: hypothesis on the MH370 flight crash site

One of the possible applications of the results of our work involves the reconstruction of Lagrangian trajectories backward in time. The concept behind this application is to discover the ocean region where a floating object, given its spatial coordinates at a certain time, could come from. Regarding this, we showed how a ’minimal’ amount of information could be sufficient to obtain a result compatible and complementary to other independent sources.

The disappearance of the Malaysia Airlines Kuala Lumpur-Beijing MH370 flight on March 8th 2014 can be classified as one of the greatest mysteries in aviation history. We recall here a brief summary of data and facts, publicly available, concerning the MH370 case:

- On March 8th, 2014, at 00:42 (Malaysia Time, MYT) Flight 370 took off from Kuala Lumpur airport.
- Final verbal contact with air traffic control occurred at 01:19 (MYT).
- Last known location, near the limits of Malaysian military radar, was at 02:22 (MYT), over the Andaman Sea.
- At 02:25 (MYT) the aircraft’s satellite communication system sent a log-on request which was received by the Inmarsat geostationary satellite orbiting at 64.5°E over the equator.
- After logging on to the network, the aircraft responded to hourly status requests from the Inmarsat for seven times, the last of which occurred at 08:10 (MYT).
- At 08:19 (MYT) the aircraft sent another log-on request, the last one.
- The aircraft did not respond to a status request from Inmarsat at 09:15.
- No trace of debris was found until July, 29^*th*^ 2015, when a flaperon, later officially recognized as part of the missing airplane, was recovered at Reunion Island.
- Successively, a total of 24 marine debris items were recovered, part of which were classified as likely related to the missing airplane.

On the basis of these data, a search area was defined considering the 7^*th*^ BTO arc corresponding to the distance from satellite of Flight 370 at the time of its last transmission, i.e. at about 4750 km on the Earth surface from point 0°, 64.5°E. After the last transmission to Inmarsat, the aircraft may have flown in any direction for about one hour before disappearing in the ocean. This implies a maximum additional ~500–1000 km uncertainty error around the 7^*th*^ BTO arc. BFO satellite data, also, give indications about the airplane heading and speed. Another useful information comes form the estimated maximum flight range from the last known position detected by military radars over the Andaman Sea, which gives a circular area of radius about 4800 km, centered at about 6°N, 96°E. Uncertainty errors on the spatial distances here considered are assumed to be (at least) ~5%.

We will see now how to potentially relate ocean drifter data to the search for the impact area of the aircraft in the ocean. SVP drifter trajectories are assumed as proxy of the unknown path followed by the debris across the marine waters. Undrogued drifters, namely, can be considered a good approximation of reconstructed flaperon-like floating objects in the ocean (see Griffin *et al*. The search for MH370 and ocean surface drift, report n. EP167888, CSIRO Oceans and Atmosphere, Dec. 8th, 2016, for these and many other details).

Since arbitrarily close Lagrangian trajectories tend to separate in time, more than one drifter need to be considered. From the NOAA Global Drifter Program data set, we selected 4 drifter tracks responding to the following requirements: passing by Reunion Island at no more than 50 km and lasting at least 508 days. Essential data describing the selected drifters are reported in Table 2. It is interesting to notice that all 4 drifters lost their drogue soon after their release. This make them good candidates to approximate the motion of a flaperon-like object floating on the ocean surface.

It must be stressed that the drifter tracks are shifted in time between one another and are not contemporary to the period of the disaster. Furthermore, we assumed that Lagrangian transport over long times and large scales, being an integral quantity, is not extremely sensitive to the details of the ocean circulation. In support of this hypothesis we observe that statistical quantities characterizing the Lagrangian phase space, e.g. the FSLE, do not depend strongly on the details of the Eulerian fields but, rather, on the relationship between the spatial and temporal scales of the flow. Were this not true, chaotic or turbulent flows could not even be analyzed from a statistical point of view. We also assumed that the time gap between the discovery of the debris and its actual arrival at the island was not very large (as reported, the debris was discovered by personnel in charge of the cleaning of the shores).

**Figure 6:**
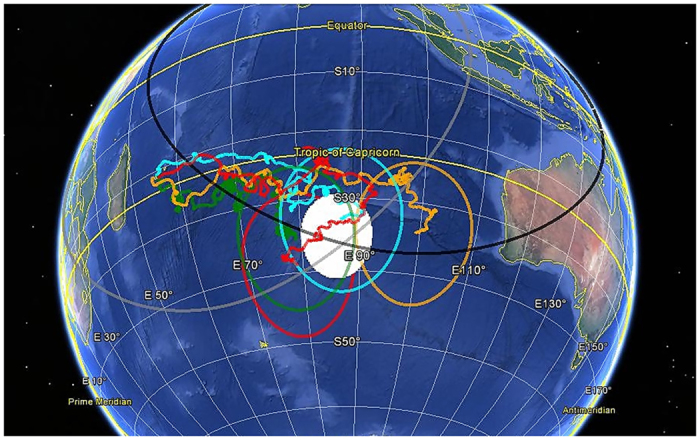
Estimate of MH370 impact area in the Southern Indian Ocean. The hypothetical impact region is the white shaded circular area centered in −35°S, 86°E. Drifter identification numbers are: 24746 (red), 70854 (orange), 70969 (green) and 83341 (cyan). Localization errors associated to each ending point of the drifters are the circular areas of radius ~10^3^ km of the same color as the corresponding drifters. The gray circle represents the 7^*th*^ BTO arc measured from Inmarsat of radius ≃ 4750 km centered in 0°, 64.5°E. The black circle represents the maximum flight range from the last known position of MH370 of radius ≃ 4800 km centered in 6°N, 96°E. This map was generated with free online utility GPS Visualizer (http://www.gpsvisualizer.com/) and Google Earth (https://www.google.com/earth/).

The estimate of the impact area is graphically illustrated in Fig. 6. The actual motion of the 4 drifters is towards Reunion Island and takes 508 days to reach the closest approach point. We assume that the 4 drifter trajectories are an approximation of the unknown path followed by the debris before arriving at the Reunion Island and, therefore, a time evolving error must be associated to each of them.

From the SIstg FSLE results (Fig. 2) we can reconstruct the mean error to associate with the predicted final positions. Considering the characteristic parameters related to the dispersion process (Fig. 5), we identified three regimes: exponential growth, with mesoscale Lyapunov exponent 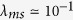
1/day at 1–30 km; Richardson law, with turbulent dissipation rate *ε* ≃ 10^−9^ m^2^ s^−3^, at 30–300 km; diffusive growth, with eddy diffusion coefficient *K*_*E*_ ≃ 10^4^ m^2^ s^−1^, up to 1000 km. The computation of the final error size amounts to *σ*_*E*_ ≃ 10^3^ km. After 508 days the error growth entered the diffusive regime, therefore we assumed a normal error distribution with variance 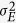. Noticeably, the relative distance between the drifters was of the same order as that of *σ*_*E*_.

Finally, we extracted a probabilistic estimate of the impact area as the product of the 4 disjoint normal distributions. This is a circular region of ≃500 km radius, centered approximately at −35°S, 86°E, and overlapping with the southernmost side of the search area (http://atsb.gov.au). In Fig. 6 the white shaded circular area represents the width of the intersection pdf at half the peak value. It is worth noticing that the white spot, as well as the error areas associated to the ending points of the 4 drifters, appear to be compatible with the bounds defined by the 7^*th*^ BTO arc and the estimated maximum flight range before ending somewhere in the ocean.

Our estimate of the impact area is in fair agreement with numerical simulations performed with an Ocean Drift Model at International Pacific Research Center (http://iprc.soest.hawaii.edu/news/MH370_debris/IPRC_MH370_News.php), in which a wide range of critical parameters characterizing the wind effects on the floating object were considered (N. Maximenko, personal communication).

At the end of this section, we would like to remark that the main goal of this work was to analyze and discuss the relative dispersion properties in the ocean surface layer, and not specifically to address the extremely difficult task of solving the mystery of the MH370 crash site. Other approaches and analysis strategies are currently under consideration, concerning all possible flight paths compatible with the satellite BTO and BFO data sequences, different debris sites, drift model simulations, temperature of the water and presence of barnacles on the debris, etc., which could possibly lead to different conclusions (R. Godfrey, personal communication). Unfortunately, we fear that the great amount of time spent unsuccessfully so far is not at all promising for the future of the search operations.

## Methods

In this paper we refer to relative dispersion, i.e. to statistics based on particle pair separation, which provides, in principle, information at any scale of motion. We considered the trajectory evolution of a Lagrangian particle, passively transported by the flow, given by the equation:


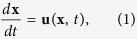


where **x** is the spatial coordinate vector, *t* is the time variable and **u** is a sufficiently regular velocity field, a perturbation along a trajectory is defined as the difference between two initially close trajectories


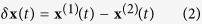


with ||*δ***x**(0)|| → 0. The norm ||⋅||, of course, depends on the geometry of the space where the flow is defined. As long as the size of the perturbation remains *infinitesimal*, i.e. 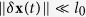, where *l*_0_ indicates the characteristic length of the Eulerian features, its time evolution can be linearized as given in the equation below:


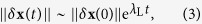


where *λ*_*L*_ is called maximum Lagrangian Lyapunov Exponent, or LLE^31^. Lagrangian chaos^32^ occurs, by definition, if *λ*_*L*_ > 0. The necessary conditions for this property to hold are that the variable *u* must be a non linear function of the spatial coordinates, with also explicit time dependence in the case of 2D fields. In a regime of Lagrangian chaos, the distance between two initially close particles grows, on average, exponentially fast with the time. The exponential growth is normally damped for significantly large separation scales, and it is generally replaced with some power law, depending on the type of diffusive regime experienced by the trajectories:


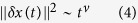


where *ν* = 1 represents standard Taylor diffusion^13^, i.e. the regime in which trajectories separate from each other as in a random walk process; *ν* = 2 describes ballistic or shear dispersion^33^, i.e. when a non zero mean velocity difference persists between two trajectories as in a constant shear flow; *ν* = 3 corresponds to Richardson diffusion^5^, i.e. a super-diffusive regime characterizing turbulent fluids, in which the diffusivity scales with the trajectory separation according to the celebrated “4/3” law, etc.

### The Finite-Scale Lyapunov Exponent

The direct measure of the time behavior of the relative dispersion hides some drawbacks that may alter the results. In particular, the fixed-time average of the inter-particle distance over an ensemble of particle pairs may be affected by *scale interference*. This occurs when particle pairs belonging to substantially different dispersion regimes are averaged together, e.g. one pair in an exponential phase (small-scale separation) and, at the same time, another pair in a diffusive phase (large-scale separation). To address this problem, considering relative dispersion as a physically scale-dependent process, one can adopt a different approach based on a properly defined scale-dependent indicator. This indicator is known as Finite-Scale Lyapunov Exponent (FSLE) and was formerly introduced in the framework of the dynamical systems theory as an extension of the concept of Lyapunov exponent^34^,^35^. The FSLE analysis was first applied to oceanographic Lagrangian data in a study of drifter dispersion in Adriatic Sea^36^ and, later, adopted by several authors in various Lagrangian dispersion studies^6^,^37^. This technique was employed, also, to define stirring rate maps and to highlight the presence of dynamical barriers to transport and mixing of tracers^38^, and to measure the sensitivity of a system to uncertainties in the evolution equations, in so-called second-kind predictability studies in the presence of macroscopic (i.e. not infinitesimal) perturbations^28^,^39^,^40^.

The FSLE is defined as follows:


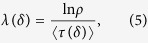


where 〈*τ(δ*)〉 is the mean time taken by the distance between two trajectories to grow from *δ* to *ρδ*, and *ρ* > 1 is a fixed growth ratio. Essentially, the FSLE gives the spectrum of relative dispersion rates as function of the spatial scale. Keeping *ρ* ~ *O*(1) guarantees that contributions from different scales of motion are not averaged together. The statistics of *τ(δ*) is obtained by considering the first exit times from *δ* to *ρδ* over a large number of realizations. Operationally, one defines a range of *N* scales *δ*_*n*_ = *ρδ*_*n*−1_, with *n* = 1, …, *N*, and for each *δ*_*n*_ one computes the mean growth time *τ(δ*). The major advantage of this approach is that one has a tool that measures the evolution of a trajectory perturbation, i.e., in Lagrangian terms, the relative dispersion process, on any scale of motion, and that returns back physically consistent estimates of the parameters that describe a given dispersion regime. The properties of the FSLE are therefore used to characterize the relative dispersion process in the ocean surface layer in terms of scaling laws and physical indicators. It is useful to recall the expected scaling laws associated with the most common dispersion regimes:

- *λ(δ*) ~ *ε*^1/3^ · *δ*^−2/3^, for Richardson turbulent diffusion, where *ε* is the mean turbulent dissipation rate appearing in a *k*^−5/3^ Kolmogorov-type of spectrum^41^,^42^;
- *λ(δ*) ~ *K*_*E*_ · *δ*^−2^, for Taylor standard diffusion^13^, where *K*_*E*_ is the eddy diffusion coefficient;
- *λ(δ*) ~ *s* · *δ*^−1^, for ballistic or shear dispersion^33^, where *s* is the mean separation velocity between two trajectories; in the context of the present work, the term shear is not meant as vertical shear, but in a broad sense as a type of relative dispersion that is characterized by a constant velocity difference within some scale range;
- *λ(δ*) ~ *δ*^−1/2^, for turbulent dispersion in the presence of a *k*^−2^ type of spectrum^43^;
- *λ(δ*) ~ constant, for exponential separation in the presence of a *k*^−3^ type of spectrum or steeper^44^, or simply due to non-local chaotic advection^31^.

We remark that the measure of turbulent dissipation rate, mean shear velocity, eddy-diffusion coefficient or Lyapunov exponents is given by the fitting parameters of the scaling laws that approximate the FSLE behavior. For example, if in some range the FSLE scales as *C*_*fit*_ · *δ*^−2^, the fitting constant *C*_*fit*_ gives a direct estimate of the eddy-diffusion coefficient 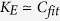, see point b) above, except order O(1) factors. Analogously, in a Richardson regime where the FSLE scales as *C*_*fit*_ · *δ*^−2/3^, the estimate of the turbulent dissipation rate is given by 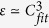, see point a) above, except order O(1) factors, and so on. In the case of systems characterized by strong anisotropy, e.g. the polar jet stream in stratosphere, the Antarctic Circumpolar Current in the Southern Ocean or experimental flows generated in a rotating tank, the FSLE can be split into components in order to separate the contributions to the dispersion coming from along-stream and cross-stream directions^45^,^46^,^47^. The time evolution of the mean distance between two trajectories, if needed, can be reconstructed from the FSLE in the following way. We know that, by definition, 〈*τ(δ*_*n*_)〉 = ln *ρ/λ(δ*_*n*_) is the mean time taken by *δ*_*n*_ to grow a *ρ* factor and become *δ*_*n*+1_. The total mean growth time from *δ*_0_ to *δ*_*n*_ is therefore:


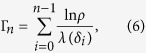


for *n* ≥ 1. This quantity can be used to estimate, for example, the size of the spreading of a tracer, initially concentrated on a scale *δ*_0_ at *t* = 0, after a given time *t* = Γ_*n*_. In other words, from the FSLE analysis one can extract a series of size-time coordinates (*δ*_*n*_,Γ_*n*_) that allow association of an error to the position of a tracer along a control trajectory (see application to the MH370 case previously discussed).

### Details of data analysis

In this paper we analyzed Surface Velocity Program (SVP) drifter data from the NOAA Global Drifter Program (GDP)^10^. We used the entire database available, last updated in August 2016, containing 20724 buoys divided in four files:

buoydata_1_5000.dat, buoydata_5001_10000.dat, buoydata_10001_15000.dat and buoydata_15001_jun16.dat.

For each buoy the files report the ID (identification number), month, day (with a typical resolution of 0.25 days), year, latitude, longitude, temperature, zonal velocity, meridional velocity, speed, variance of latitude, and the variance of longitude. A label controlling the presence of the drogue (1 = drogue on, 0 = drogue off) at every time step is further added to the analysis. Drifter data were first divided by geographical area according to the ocean partition shown in Fig. 1. Then, for each ocean sub-basin, all available simultaneous trajectory pairs were prepared for the FSLE analysis reported in Fig. 2. The maximum initial shell for the drifter separation was fixed at Δ = 10 km. This means that any initial separation *δ* ≤ Δ is considered acceptable. The scale density factor was set as 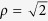. Error bars on the FSLE are estimated as the standard deviation on the mean value, which by definition is 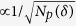 where *N*_*p*_(*δ*) is the number of drifter pairs counted in the statistics at given separation scale *δ*, see Fig. 3. In all results, only statistical points with *N*_*p*_(*δ*) > 5 are plotted.

Additional FSLE analysis is also presented for GPS-tracked drifter data, Fig. 4. We considered the driftertrajGPS.txt datafile containing hourly location and velocity of GPS surface drifters from the GDP (ftp://ftp.aoml.noaa.gov/pub/phod/buoydata/hourly_product/v1.00/). The file contains one hourly data entry per line, with the following 9 columns: identification number from the GDP, Julian day since 01/01/1979, hour of day (UTC), longitude, latitude, zonal velocity, meridional velocity, length of interpolating gap in hours, drogue status: 1 = drogue on, 0 = drogue off. Only drogued drifter pairs (1,1) were analyzed in order to check consistency of the submesoscale results obtained from the ARGOS-tracked drifter data. Location errors of GPS-tracked drifter data are reported of order 10^−5^ degree.

Sensitivity of the FSLE measure to various combinations of Δ and *ρ* was also checked out and reported in Supplementary Material, Fig. S2. The results turned out to be very stable for a significantly large range of variation of these parameters.

## Additional Information

**How to cite this article**: Corrado, R. *et al*. General characteristics of relative dispersion in the ocean. *Sci. Rep.* **7**, 46291; doi: 10.1038/srep46291 (2017).

**Publisher's note:** Springer Nature remains neutral with regard to jurisdictional claims in published maps and institutional affiliations.

## Supplementary Material

Supplementary Materials

## Figures and Tables

**Table 1:** List of acronyms.

NPspg	North Pacific sub-polar gyre
NS	Nordic Seas
NPstg	North Pacific sub-tropical gyre
NAstg	North Atlantic sub-tropical gyre
EIcs	Equatorial Indian current system
EPcs	Equatorial Pacific current system
EAcs	Equatorial Atlantic current system
SIstg	Southern Indian sub-tropical gyre
SPstg	Southern Pacific sub-tropical gyre
SAstg	Southern Atlantic sub-tropical gyre
SO	Southern Ocean
ACC	Antarctic Circumpolar Current
NOAA	National Oceanic and Atmospheric Administration
GDP	Global Drifter Program
SVP	Surface Velocity Program
ARGOS	Advanced Research and Global Observation Satellite
GPS	Global Positioning System
LLE	Lagrangian Lyapunov Exponent
FSLE	Finite-Scale Lyapunov Exponent
BTO	Burst Time Offset
BFO	Burst Frequency Offset

**Table 2:** Identification numbers and dates of release, end of run and loss of drogue of the drifter tracks reported in Fig. 6.

Id. number	Release	End of run	Loss of drogue
24746	9/21/2001	7/24/2004	2/13/2002
70854	9/4/2007	11/15/2009	9/22/2007
70969	10/10/2010	9/7/2013	10/14/2010
83341	11/06/2008	5/4/2012	12/16/2008
