# Supplementary material for: General characteristics of relative dispersion in the ocean
Source: Sci Rep. 2017 Apr 11;7:46291. doi: 10.1038/srep46291 (PMC5387742; doi:10.1038/srep46291)
Supplement: Supplementary Materials [file srep46291-s1.pdf]

Supplementary Material to the manuscript
General characteristics of relative dispersion in the Ocean
R. Corrado, G. Lacorata, L. Palatella, R. Santoleri and E. Zambianchi

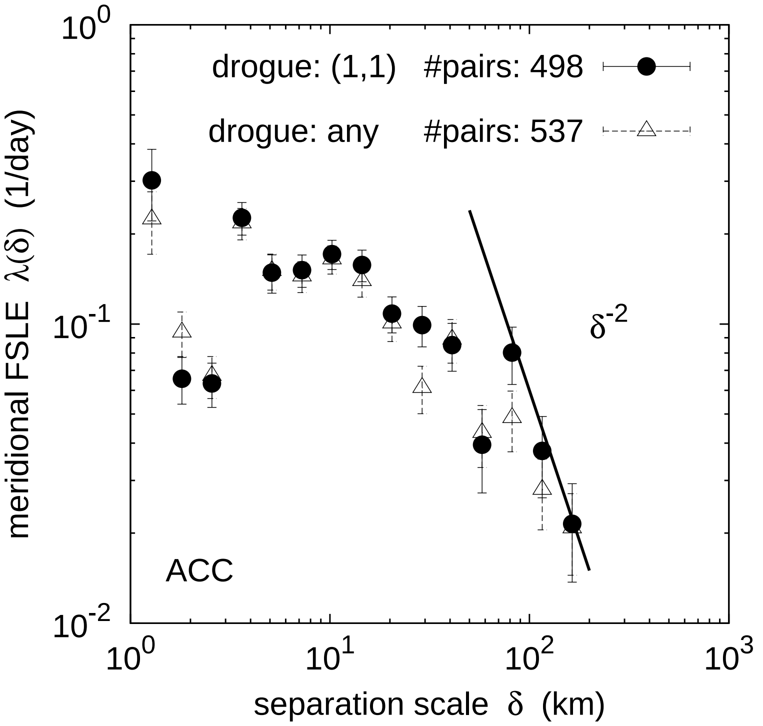

Fig. S1: Meridional FSLE for Southern Ocean drifter pairs. Only two cases are reported: pairs with drogued drifters (1,1), and pairs of drifters regardless their drogue status. The δ^{-2} scaling, for separations of order 100 km, corresponds to a cross-stream eddy-diffusion with a meridional eddy-diffusivity estimated as $\simeq 5 \cdot 10^3$ m^{2}s^{-1}.

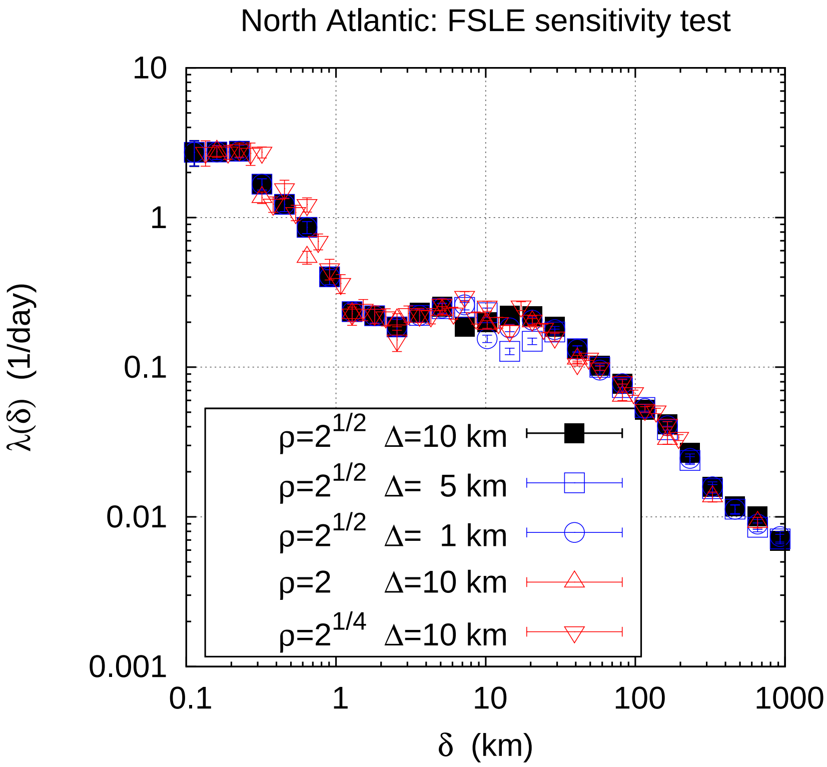

Fig. S2: Sensitivity analysis of the FSLE to parameter change: ρ is the amplification factor between neighbouring scales, and Δ is the maximum initial shell allowed for the FSLE computation. Data used for the test refer to the North Atlantic Ocean. The values $\rho = 2^{1/2}$ and $\Delta = 10$ km (full black squares) are those used in the FSLE analysis discussed in the paper. As can be seen, the shape of the FSLE curve is rather stable to significantly large parameter variations.
